# Quantifying telomere length: from bulk assays to single-molecule resolution

**DOI:** 10.52601/bpr.2025.240068

**Published:** 2026-06-30

**Authors:** Kangkang Ma, Zhongbo Yu

**Affiliations:** 1 State Key Laboratory of Medicinal Chemical Biology, College of Pharmacy, Nankai University, Tianjin 300350, China

**Keywords:** Telomere length, Terminal restriction fragments, Nanopore, Magnetic tweezers, Single-molecule

## Abstract

Telomere length (TL) is a promising biomarker for age-associated diseases and cancer. Single-molecule studies of human TL are advancing rapidly, providing unprecedented insights into the dynamics and variability of telomeres at the single-molecule level. TL is commonly reported as average TL or relative TL, depending on the methods used. However, average TL, short TL, and long TL have distinct significances: average TL serves as a general biomarker for aging, short TL indicates the risk of age-related diseases, and long TL is associated with certain cancers and all-cancer mortality. Thus, the TL distribution is more important than the average TL alone. Single-molecule techniques measure TL one telomere at a time, offering quantitative TL distributions that are crucial for understanding telomere biology. In this review, we focus on various TL measurement techniques, with particular emphasis on single-molecule methods. Single-molecule studies of human TL are poised to play a pivotal role in advancing our understanding and clinical management of age-associated diseases and cancer.

## INTRODUCTION

**Figure 1:**
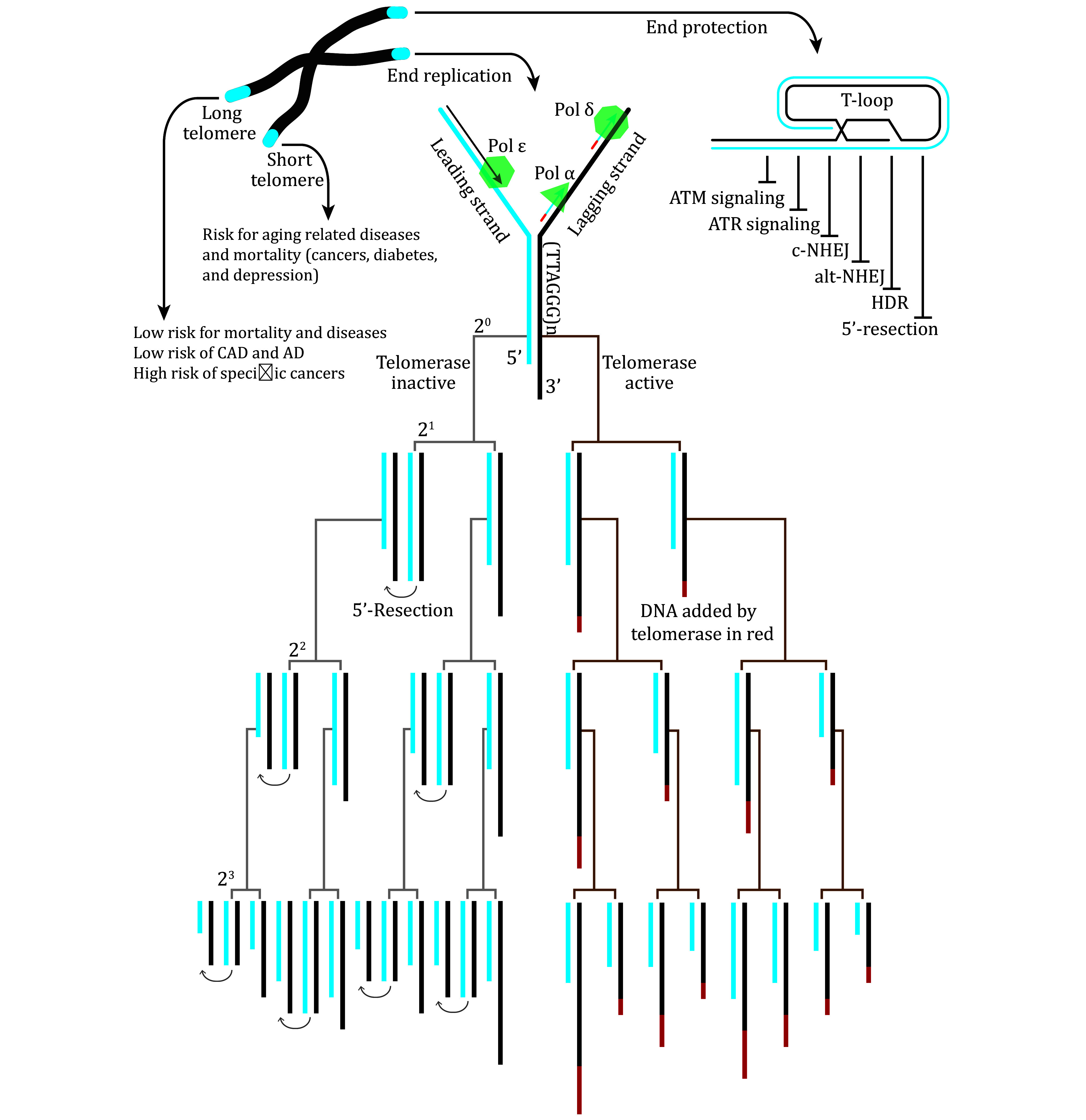
Human telomeres and their length homeostasis. Human telomeres at the ends of chromosomes are illustrated, along with the end-replication and end-protection problems. During telomere replication, the C-rich 5'-ended strand serves as the template for leading-strand DNA synthesis, while the G-rich TTAGGG repeat strand serves as the template for lagging-strand DNA synthesis. The replication products are shown, with the leading-strand replication products undergoing 5′-resection. Telomerase adds sequences to the 3′-overhang of the replication products. T-loop structures and the shelterin complex protect telomeres from DNA damage responses. The resulting short and long telomeres are associated with human aging and various diseases

Human linear chromosomes end at telomeres, which consist of repetitive TTAGGG DNA sequences and form a unique chromatin structure distinct from normal nucleosomes (Bandaria *et al.* 2016; Hanish *et al.* 1994; Meyne *et al.* 1989; Soman *et al.* 2022; Yu *et al.* 2012). Telomeres face two major challenges: the end-replication problem (incomplete replication of linear DNA ends) and the end-protection problem (prevention of telomere recognition as DNA damage). These issues are mechanistically linked to shelterin-mediated telomere maintenance (Bejarano *et al.* 2019; de Lange 2018; Hayashi *et al.* 2015) and enzyme recruitment (Brenner and Nandakumar 2022; Cai and de Lange 2023; Cai *et al.* 2024). As cells divide, telomeres gradually shorten due to the end-replication problem (Karimian *et al.* 2024; Sholes *et al.* 2022; Takai *et al.* 2024). Once telomeres reach a critically short length, the cell enters a state of replicative senescence, halting further division. This is a physiological mechanism that can prevent cancer development, and the pathological processes involved are due to the telomere end-protection problem, which triggers DNA damage responses (DDR) (Galli *et al.* 2021; Hewitt *et al.* 2012; Myler *et al.* 2023). This cellular aging process also contributes to tissue dysfunction and the decline in regenerative capacity over time, leading to many age-related diseases, such as cardiovascular disease, neurodegenerative disorders, and cancer (Fig. 1) (Bao *et al.* 2023; Blackburn *et al.* 2015; Maciejowski and de Lange 2017; Myllymäki *et al.* 2020).

Telomere length (TL) homeostasis involves the balanced action of telomerase, which extends the G-rich strand and maintains the 3'-overhangs, and the CST-Polα-primase complex, which ensures proper replication of the C-rich strand during lagging-strand synthesis, to prevent progressive telomere shortening and maintain chromosomal stability. The end-replication problem is the inherent challenge of replicating the very ends of linear chromosomes (Cristofari and Lingner 2006; Teixeira *et al.* 2004; Vodenicharov and Wellinger 2007; Weuts *et al.* 2012). Telomerase acts after DNA replication to extend both the leading- and lagging-strand DNA synthesis products, maintaining the 3'-overhangs (Parks and Stone 2017). The incomplete duplication of the C-rich strand by lagging-strand DNA synthesis is resolved by the CST-Polα-primase complex through fill-in synthesis to replenish the lost sequences at the 5'-ends of the lagging-strand product. Telomerase levels are limited and preferentially act on shorter telomeres, and a switch between extendible and non-extendible states ensures stable TL over multiple cell divisions. E. H. Blackburn noted that shelterin mediates telomerase action in TL homeostasis (Blackburn 1997; Liu *et al.* 2021).

Shelterin is a critical complex composed of six proteins (TRF1, TRF2, POT1, TIN2, TPP1, and RAP1) that protect telomeres from various DDR mechanisms and ensure their stability (Erdel *et al.* 2017; Lin *et al.* 2014; Sfeir and de Lange 2012). TRF2 plays a pivotal role by wrapping approximately 90 base pairs of telomeric DNA through lysine and arginine residues localized around its homodimerization domain (Benarroch-Popivker *et al.* 2016). This unique DNA wrapping mechanism forms and stabilizes the t-loop structure, which is essential for inhibiting the activation of the ATM kinase and the nonhomologous end-joining (NHEJ) pathway. Furthermore, TRF2's basic domain binds to branched DNA, such as t-loop junctions, preventing cleavage by Holliday junction resolvases and masking the binding site for PARP1, thereby safeguarding telomere integrity (Schmutz *et al.* 2017). TRF2 also mediates the columnar stacking of human telomeric chromatin (Wong *et al.* 2024). TRF1, another key component of shelterin, promotes efficient replication of the TTAGGG repeats by preventing replication fork stalling (Bianchi *et al.* 1997; Sfeir *et al.* 2009). This function is crucial for overcoming the challenges posed by the repetitive nature of telomeres, which can otherwise lead to replication-dependent defects like those observed at fragile sites.

In addition to TRF1 and TRF2, the shelterin complex includes TPP1 and POT1, which are essential for telomerase recruitment and activation (Kibe *et al.* 2016; Liu *et al.* 2022). TPP1 interacts with the N-terminal domain and the RAP motif of telomerase reverse transcriptase, stabilizing these interactions and dampening conformational dynamics, thus defining the requirements for telomerase recruitment and activation. TIN2, which binds to both TRF1 and TRF2, is crucial for stabilizing the TPP1/POT1 complex on single-stranded telomeric DNA, preventing the binding of RPA and repressing ATR signaling (Takai *et al.* 2011). The loss of RAP1, although not essential for TRF2's primary functions, is critical for repressing homology-directed repair (HDR), which can alter TL (Sfeir *et al.* 2010). Telomeric repeat-containing RNA (TERRA) facilitates heterochromatin formation and the recruitment of the origin recognition complex to telomeres, further contributing to telomere structural maintenance (Deng *et al.* 2009).

Telomeres are often recognized by the cell as DNA double-strand breaks (DSBs), which activate the DDR pathway (Hewitt *et al.* 2012; Kinzig *et al.* 2024). This pathway, primarily mediated by the ATM and ATR kinases, is essential in aging and senescent cells, where telomere dysfunction is more prevalent (Lee *et al.* 2015). The DDR detects and responds to telomere shortening and damage, preventing genomic instability and cellular senescence. Stalled replication forks, a common issue during DNA replication, increase telomerase recruitment in an ATR-dependent manner (Tong *et al.* 2015). Additionally, the phosphorylation of TRF1 by ATM/ATR kinases leads to its loss from telomeres, potentially increasing replication fork stalling and further activating the DDR (Takasugi *et al.* 2023). In the context of the alternative lengthening of telomeres (ALT) pathway, used by some cancer cells and immortalized cell lines to maintain TL without telomerase, the BLM helicase plays a crucial role (Silva *et al.* 2022). BLM unwinds lagging-strand telomere intermediates, facilitating the assembly of a replication-associated DDR at ALT telomeres (Jiang *et al.* 2024; Zhao *et al.* 2019). This function is essential for resolving replication stress and maintaining telomere stability. TERRA forms RNA:DNA hybrids (R-loops) at ALT telomeres and interacts with various DNA repair proteins (Decottignies 2022; Yadav *et al.* 2022). One such protein, the endonuclease XPF, is highly enriched at ALT telomeres and is recruited by telomeric R-loops to induce the DDR independently of CSB and SLX4 (Guh *et al.* 2022). The activation of DDR by XPF triggers break-induced telomere synthesis and lengthening, promoting homologous recombination and telomere replication (Yang *et al.* 2020). The m6A modification of TERRA, mediated by METTL3, further stabilizes these R-loops, and the m6A reader YTHDC1 facilitates their formation, ensuring telomere stability in ALT cells (Chen *et al.* 2022).

The methods for measuring TL have been evolving and are reviewed elsewhere (Coulter *et al.* 2024; Ferrer *et al.* 2023; Lai *et al.* 2018; Lindrose *et al.* 2021; Yu *et al.* 2024). TL is commonly reported through the average TL or relative TL according to the methods used. However, average TL, short TL, and long TL have distinct significances: average TL serves as a general biomarker for aging, short TL indicates the risk of age-related diseases, and long TL is associated with certain cancers and all-cancer mortality. Therefore, the TL distribution is more important than the average TL alone. Single-molecule techniques measure TL one telomere at a time and provide quantitative TL distributions, which are helpful in understanding telomere biology (Mazzucco *et al.* 2022; Nai *et al.* 2024). In this review, we focus on TL measurement techniques (Table 1), with particular attention to single-molecule methods.

**Table 1:** Key features of TL methods

Method	Sample input	Resolution	Throughput	Key advantages	Main limitations
TRF (Allshire *et al.* 1988; Kimura *et al.* 2010; Mender and Shay 2015)	>1 μg DNA or >10^5^ cells	~1 kb	~130 samples/week	Gold standard, absolute TL	High DNA input, bulk average, TL overestimation
qPCR (Cawthon 2009; Martin *et al.* 2024)	~10 ng DNA	NA	96 samples	Rapid measurements, high-throughput, low cost	High variability, reference-dependent
Digital qPCR (STAR) (Luo *et al.* 2020)	<1 ng DNA	~0.2 kb	48 samples	Rapid measurements, absolute TL	Specialized equipment, expensive chips
ΩqPCR (Xiong and Frasch 2021)	Single cells	20–40 bp	96 samples	Single-cell measurements, absolute TL	Technically complex, requires specialized optimization
STELA (Baird *et al.* 2003)	0.10–0.25 ng DNA	0.1–0.5 kb	Lower than TRF	Single-telomere measurement, chromosome-specific TL	Technically challenging, primer design difficulties, PCR variability
U-STELA (Bendix *et al.* 2010)	10–40 pg DNA	0.1–0.5 kb	Lower than TRF	Single-telomere measurement, genome-wide short TL analysis	Complex workflow, PCR variability, biased against long TL
TeSLA (Lai *et al.* 2017)	20–40 pg DNA	0.1–0.5 kb	Lower than TRF	Single-telomere measurement, shortest TL detection	Complex workflow, input DNA dependent PCR variability
Q-FISH (Canela *et al.* 2007; Fouquerel and Opresko 2020; Lansdorp *et al.* 1996; Nonaka *et al.* 2025)	30,000–90,000 cells	~0.5 kb	96 samples	Single cell measurements, chromosome-specific TL	Requires metaphase cells, small data sets
Flow FISH (Baerlocher *et al.* 2006; Wand *et al.* 2016)	100,000–900,000 cells	~0.5 kb	22 samples/3 days	High-throughput, cell population analysis	Technically demanding, relative quantification, requires fresh cells
DNA-array-FISH (Zheng *et al.* 2024)	0.5–1 μg DNA	~0.1 kb	96 samples	Large data sets, large TL scale	Standard curve dependent, expensive chips
Nanopore (Karimian *et al.* 2024; Sanchez *et al.* 2024)	40 μg DNA	30 bp	8 samples	Single-telomere measurement, chromosome-specific TL	Computational complexity, reference genome dependency
Magnetic Tweezers (Li *et al.* 2021)	0.5 μg DNA	<10 bp	8 sample/day	Single-telomere measurement, reference independent	Specialized equipment, requires surface immobilization

## TRF: THE GOLD STANDARD FOR TL ANALYSIS

Average TL can be indirectly measured using Telomere Restriction Fragment (TRF) analysis. TRF is a modified Southern blot technique that assesses telomere length by analyzing the length distribution of terminal restriction fragments. The principle of TRF relies on the absence of restriction enzyme recognition sites within the telomeric TTAGGG tandem repeats. Therefore, when genomic DNA is digested with a combination of 4-base restriction endonucleases, the size of the genomic DNA fragments is reduced to less than 800 bp, while the telomeric DNA remains intact.

**Figure 2:**
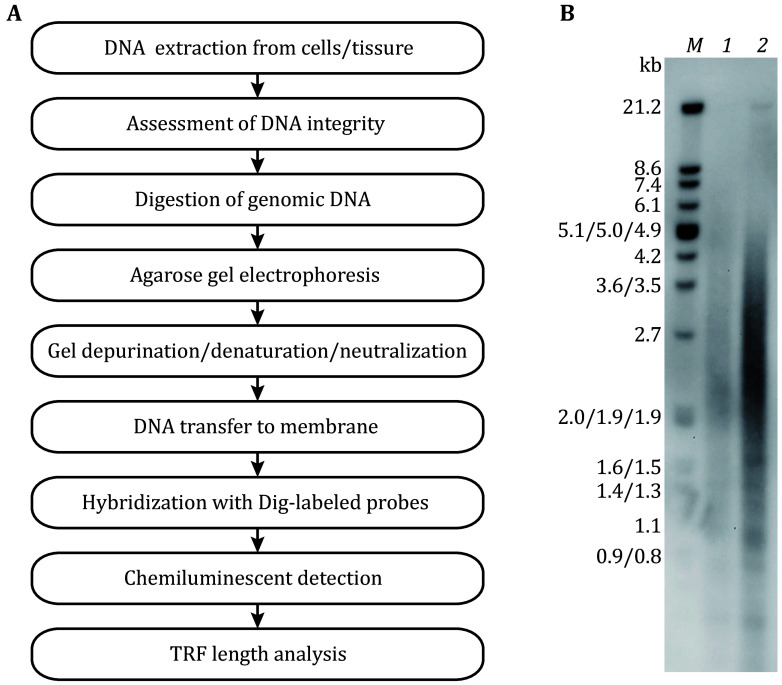
Procedure and typical results of TRF for TL analysis. **A** The representative procedure for TRF analysis is illustrated. **B** A typical TRF result using K562 cells is shown. Panel A was adapted from the reference Kimura *et al.* (2010). Panel B was adapted from the reference Li *et al.* (2021)

TRF is widely regarded as the gold standard for quantitative TL measurement and has been extensively reviewed and described in detail elsewhere (Harley *et al.* 1990; Kimura *et al.* 2010; Mender and Shay 2015). In brief, the TRF method involves the following steps (Fig. 2): DNA extraction and integrity inspection, digestion with restriction enzymes, separation via multiplexed gel electrophoresis, and transfer to a membrane for hybridization with labeled probes for imaging. This robust method measures the average TL of all telomeres in a sample, providing a global view of telomere dynamics, which is particularly valuable for comprehensive analysis.

Historical data on TL using TRF is extensive, and there is a well-established protocol for data interpretation. However, TRF has several limitations. It requires a relatively large amount of DNA, approximately 3 μg per sample, and is labor-intensive, with a skilled technician capable of processing about 130 samples per week. TRF does not provide information about the length of individual telomeres, only the average length. The protocol is technically demanding and time-consuming. The presence of DNA secondary structures, such as G-quadruplexes, can affect the efficiency of restriction enzyme digestion, leading to potential artifacts in the measurement. Additionally, TRF cannot account for inter-chromosomal variability in telomere length, which may be significant in certain biological contexts. Despite these limitations, TRF remains a crucial tool in TL research, especially for developing and validating new methods to measure TL.

## THE QPCR METHODS: FROM RELATIVE TO ABSOLUTE TL QUANTIFICATION

The qPCR method is rapid and straightforward, making it accessible to technicians with basic molecular biology training. For example, TL by qPCR has been applied to 6391 tissue samples, representing over 20 tissue types and 952 individuals (Demanelis *et al.* 2020). This extensive analysis has revealed that genetic variation affects TL in multiple tissue types and that TL may mediate the effect of age on gene expression. Recently, using qPCR in conjunction with whole-genome sequencing, the genetic architecture of TL has been illustrated in 462,666 whole-genome sequences from the UK Biobank (Burren *et al.* 2024).

The qPCR method is highly sensitive and can detect TL variations with a relatively small amount of DNA, as little as 10 ng. The original qPCR method developed by R. M. Cawthon for relative TL measurement, referred to as singleplex qPCR, involves two separate reactions: one to quantify the telomere (T) signals and another to quantify a single copy gene (S) signal, typically performed in different wells or plates using two sets of primers (Cawthon 2002). R. M. Cawthon further developed the multiplex qPCR method, which overcomes several limitations of the singleplex approach (Cawthon 2009; Martin *et al.* 2024). Multiplex qPCR requires fewer reagents and is more cost-effective because it combines the quantification of both telomere and single copy gene signals within the same reaction well, thereby reducing the number of reactions needed. This also minimizes variability caused by pipetting differences, as both signals are derived from the same input DNA, enhancing the precision of the T/S ratios. Additionally, the multiplex method increases throughput and reduces the time required for analysis, as fewer reactions are needed per sample. The use of a single fluorescent DNA-intercalating dye simplifies the process, with telomere signals collected in early cycles and single copy gene signals captured after the telomere product has melted away, ensuring clear and distinct measurements for both (Cawthon 2002, 2009; Martin *et al.* 2024).

However, despite these advantages, qPCR methods, including multiplex qPCR, measure relative TL rather than absolute length, which can make it difficult to compare results across different laboratories or studies. Rigorous standardization of qPCR protocols is crucial to ensure reliable and reproducible results, but this can be challenging across different labs. Variations in PCR efficiency can affect the accuracy of TL measurements and need to be carefully controlled. Furthermore, qPCR tends to overestimate the length of very short telomeres, which can be a limitation in studies of telomere attrition or aging.

To measure absolute TL, the absolute qPCR method has been developed based on Cawthon's qPCR assay (O'Callaghan and Fenech 2011; O’Callaghan *et al.* 2008). This absolute qPCR method utilizes an oligomer standard to generate absolute TL values. Absolute TL facilitates more direct comparisons of qPCR results both within and between experiments in different laboratories, making it easier to compare findings from various research groups. Moreover, absolute qPCR shows a strong correlation with TRF analysis by Southern hybridization. However, both relative and absolute qPCR methods are still considered to be throughput limited.

To address the throughput limitation, L. F. Cheow *et al*. developed the Single Telomere Absolute-Length Rapid (STAR) assay based on a high-throughput digital real-time PCR approach. This method allows for the rapid and precise measurement of the absolute lengths and quantities of individual telomere molecules (Luo *et al.* 2020). Previously, digital PCR have been used to quantify telomerase enzyme activity at the single cell level (Ludlow *et al.* 2014; Ludlow *et al.* 2018). The high-throughput nature of digital real-time PCR in the STAR assay makes it feasible to use TL distribution as a biomarker in disease research and large-scale population studies.

Further developments in qPCR-based TL measurements include the ΩqPCR approach developed by F. Xiong and W. D. Frasch, which can determine absolute TL in kb units directly from single cells (Xiong and Frasch 2021). This method utilizes Ω-probes, which are DNA strands designed with sequence information to: (1) hybridize with the telomere via opposing 3' and 5' ends, (2) ligate the hybridized probes to form circularized Ω-probes, and (3) enable circularized-dependent qPCR through sequence information for a forward primer, a reverse primer binding site, and a qPCR hydrolysis probe binding. The accuracy and precision of ΩqPCR were validated using synthetic telomeres of 800 bp and 1600 bp inserted into plasmids, measuring 819 ± 19.6 bp and 1590 ± 42.3 bp, respectively. When combined with data on the cell cycle stage from a single-copy gene and ploidy, the average TL of single cells measured by ΩqPCR was consistent with results obtained from larger sample sizes using the traditional TRF method.

## TL METHODS BASED ON BOTH TRF AND PCR

Based on the TL principles of TRF and PCR, Single Telomere Length Analysis (STELA) was designed to measure telomeres on individual chromosomes, assessing the abundance of the shortest telomeres using a combination of ligation, PCR-based methods, and Southern blot analysis (Baird *et al.* 2003; Hills *et al.* 2009). STELA has revealed extensive allelic variation and ultrashort telomeres in senescent human cells, demonstrated telomere dysfunction and fusion during the progression of chronic lymphocytic leukemia, and accurately measured chromosome-specific TL with limited starting material. It has also been adapted to develop telomere end ligation protocols to determine the terminal nucleotides of both the C-rich and G-rich telomere strands, showing that most chromosome ends exhibit telomere extension under steady-state conditions (Sfeir *et al.* 2005; Zhao *et al.* 2009). However, the limitation of STELA is that not all chromosome ends have unique sequences for primer design, restricting the number of chromosome ends that can be monitored. To overcome this, Universal STELA (U-STELA) was introduced, capable of detecting telomeres from every chromosome end, thus enabling the monitoring of changes in the shortest telomeres (Bendix *et al.* 2010). Despite its improvements, U-STELA is not efficient at detecting TL over 8 kb, which can affect the detection and accuracy of the TL distribution, but it can still count the absolute number of the shortest telomeres.

TeSLA is another method based on the TL principles of TRF and PCR. TeSLA employs a ligation and digestion strategy, Southern blot analysis with digoxigenin-labelled probe, and image processing software to measure the distribution of telomeres at different lengths (Lai *et al.* 2017). This method allows for the measurement of the abundance and distribution of telomeres from less than 1 kb to approximately 18 kb. With TeSLA, subtle TL changes can be monitored in a short period of time, providing insights into telomere dynamics during various cellular processes. For example, TL has been measured longitudinally in peripheral blood mononuclear cells during human aging, in tissues during colon cancer progression, in telomere-related diseases such as idiopathic pulmonary fibrosis, as well as in telomerase knockout mice and other organisms. Like STELA and U-STELA, TeSLA does not measure exceptionally long telomeres, such as those in inbred strains of mice. However, for human studies, determining the shortest telomeres could have diagnostic implications for disease development (pathological thresholds) where earlier interventions may result in better patient management. Additionally, TeSLA does not amplify interstitial telomeric sequences, which consist of telomeric repeats located away from chromosome ends in vertebrates.

## QUANTITATIVE AND FLOW FISH FOR TL ANALYSIS AT THE SINGLE-MOLECULE LEVEL

Quantitative fluorescence *in situ* hybridization (Q-FISH) using formalin-fixed paraffin-embedded (FFPE) tissue sections enables the TL estimation in individual cells. In Q-FISH, fluorescently labelled peptide nucleic acid (PNA) probes hybridize to telomeric and centromeric sequences in FFPE tissue sections, and relative TL are measured by comparing telomere signal intensities to those of centromeres (Fouquerel and Opresko 2020; Nonaka *et al.* 2025). To measure the TL in specific cells, flow FISH is employed, using labeled PNA probes specific for telomere repeats and fluorescence measurements by imaging and flow cytometry (flow FISH) (Baerlocher *et al.* 2006; Sharifi-Sanjani *et al.* 2017; Wand *et al.* 2016). Flow FISH analysis can be conducted using commercially available flow cytometers. The accuracy and reproducibility of the measurements are improved by automating most pipetting steps and including an internal standard in each sample tube.

By using specific antibody staining, an optimized Q-FISH protocol enables the quantification of telomeres in individual chromosomes directly within tissue samples, thereby eliminating contributions from undesired cell types and avoiding the need for cell isolation (Sharifi-Sanjani *et al.* 2017). The main procedure involves tissue preparation, permeabilization, pretreatment and hybridization with a Cy3-labeled telomeric repeat complementing (CCCTAA)3 PNA coupled with specific antibody staining. This protocol provides cell-type-specific TL measurements in small human samples and takes ~28 h, including three overnight incubations.

Y. L. Zheng *et al*. developed the DNA-array-FISH method by combining DNA microarray technology with FISH, which measures the bp lengths of single telomeres with high throughput (Zheng *et al.* 2024). This method can analyze about 32,000 telomeres per DNA sample, and one microarray chip can handle 96 test DNA samples. Various telomere parameters, such as average TL and the frequency of short or long telomeres, are computed to characterize the TL distribution. The method demonstrates high precision, with intra-assay and inter-assay coefficients of variation for average TL ranging from 1.37% to 3.98%. Additionally, the correlation coefficient (r) for repeated average TL measurements ranges from 0.91 to 1.00, indicating excellent reproducibility. The average TLs measured by DNA-array-FISH closely correlate with those obtained by the gold standard TRF analysis, with correlation coefficients (r) ranging from 0.87 to 0.99. The ability to gather many single telomere length data points offers a unique opportunity for in-depth analysis of telomere dynamics and their complex relationship with age-related diseases.

## SINGLE-MOLECULE NANOPORE FOR TL ANALYSIS

**Figure 3:**
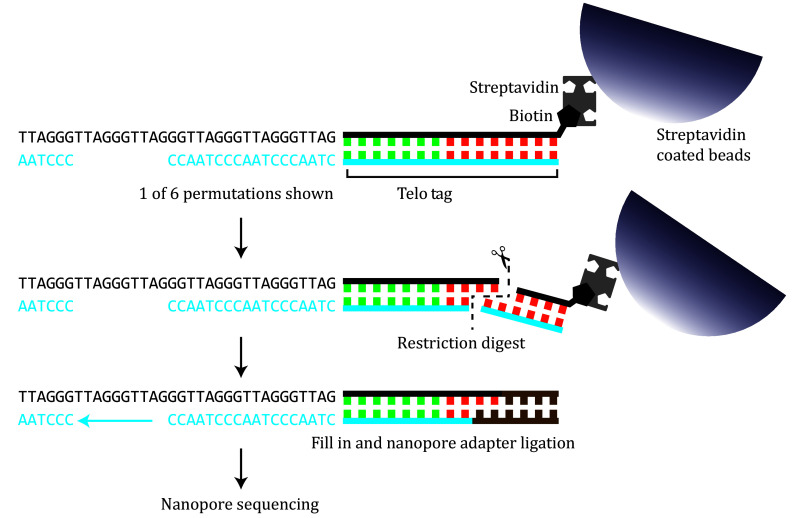
Procedure of telomere preparation for nanopore sequencing. Adapted from the reference Karimian *et al.* (2024)

Single-molecule nanopore sequencing enables direct, real-time analysis of individual DNA molecules. K. Karimian and C. W. Greider *et al*. developed a nanopore-based method called Telomere Profiling to measure TL (Karimian *et al.* 2024). Telomeres are tagged with a biotin adapter and enriched using streptavidin coated beads (Fig. 3). A single telomere then passes through a nanometer-scale pore embedded in a membrane while changes in the ionic current are detected and translated into a sequence of nucleotides. This process is enabled using nanopore sequencers like the MinION, which can generate ultra-long reads, often exceeding 100 kb. These ultra-long reads are crucial for accurately assembling telomeres, which are highly repetitive and difficult to sequence using traditional short-read sequencing methods (Kim *et al.* 2021; Schmidt *et al.* 2024). The ability to sequence and analyze these repetitive regions provides a more comprehensive understanding of TL and structure, making nanopore sequencing a powerful tool for TL analysis.

To achieve high-quality telomere-to-telomere (T2T) assemblies, researchers have developed a series of protocols that leverage the unique capabilities of nanopore sequencing (Rautiainen *et al.* 2023). One such protocol, described in the sequencing and assembly of the human GM12878 cell line, involves generating ultra-long reads with an N50 length exceeding 100 kb, using the MinION sequencer (Jain *et al.* 2018). These ultra-long reads are then combined with additional sequencing data, such as 5× coverage of shorter reads, to improve assembly contiguity. The resulting assembly has an NG50 of approximately 6.4 Mb, covering 85.8% of the reference genome with over 99.8% accuracy. Another protocol, detailed in the assembly of a complete human X chromosome, uses high-coverage ultra-long-read nanopore sequencing of the hydatidiform mole CHM13 genome, supplemented with PacBio high-fidelity reads for quality improvement (Miga *et al.* 2020; Tham *et al.* 2023). Furthermore, to address common polishing errors, a repeat-aware polishing strategy was designed, which corrected 51% of the existing errors and improved the assembly quality value from 70.2 to 73.9 (Mc Cartney *et al.* 2022).

Single-molecule nanopore sequencing has a wide range of applications in telomere research, from basic science to clinical diagnostics. One significant application is the precise measurement of TL in human cells, as demonstrated by the digital telomere measurement method (Sanchez *et al.* 2024). By using nanopore sequencing, researchers can measure telomere attrition and de novo elongation with up to 30 bp resolution in genetically defined populations of cells, blood cells from healthy donors, and blood cells from patients with genetic defects in telomere maintenance (Sanchez *et al.* 2024). This high-resolution data reveals that human aging is associated with a progressive loss of long telomeres and an accumulation of shorter telomeres. In patients with telomere biology disorders, the accumulation of short telomeres is more pronounced and correlates with the severity of the phenotype. Machine learning models trained on this data can distinguish healthy individuals from those with telomere-related diseases, advancing the use of TL as a clinical biomarker.

## SINGLE-MOLECULE TRF ANALYSIS BY MAGNETIC TWEEZERS

**Figure 4:**
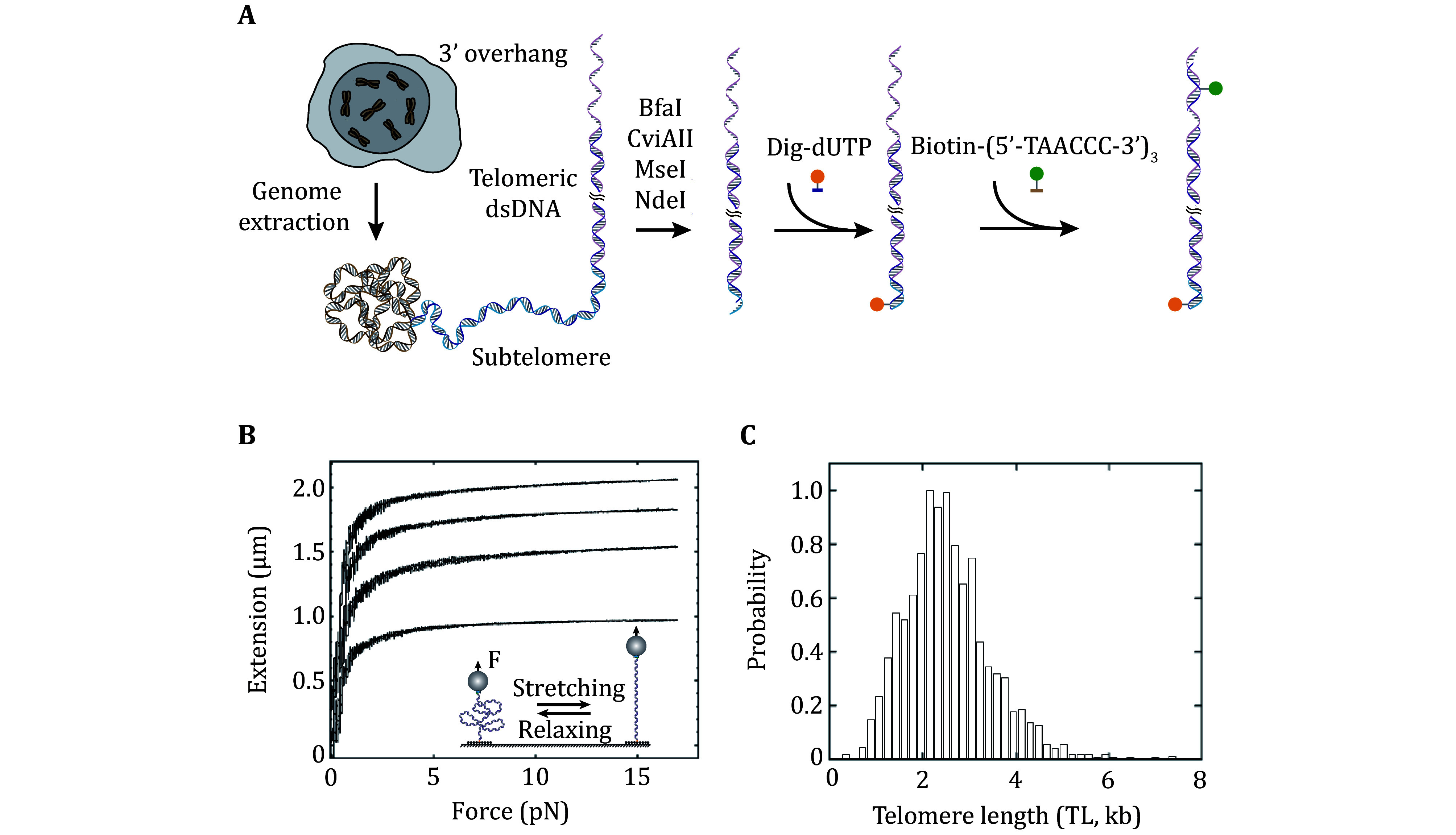
TL analysis using single-molecule magnetic tweezers. **A** The procedure for preparing telomeres for TL analysis. **B** Force-extension curves of telomeres using single-molecule magnetic tweezers. **C** TL distribution based on force-extension curves of K562 cells. Adapted from the reference Li *et al.* (2021)

We developed single-molecule TRF analysis using magnetic tweezers, which serves as a powerful technique that allows for the direct, high-resolution measurement of TL and the investigation of protein–DNA interactions at the telomeric regime (Li *et al.* 2021). Magnetic tweezers are a type of trapping system that uses magnetic forces to manipulate and measure the mechanical properties of single molecules (Gao *et al.* 2024; Li *et al.* 2019; Ma *et al.* 2024; Wang *et al.* 2023). In this method, single human telomeres are mechanically stretched and relaxed by applying controlled forces through magnetic beads (Fig. 4). The force-extension behavior of the telomeres is then recorded, and the length of individual telomeres is measured using the Worm-Like Chain (WLC) model, which describes the elastic behavior of DNA under tension. This approach provides a more precise and detailed measurement of TL compared to traditional methods such as gel electrophoresis or fluorescence analysis.

Human telomeres are isolated from K562 leukemia cells, digested with restriction enzymes (BfaI, CviAII, MseI, NdeI) to generate terminal fragments, and labeled with biotin/digoxigenin for mechanical manipulation. Enrichment via biotin probes ensures telomere specificity (>95% purity). During measurement, magnetic tweezers stretch individual telomeres attached to beads and glass surfaces, recording force-extension curves under controlled forces (±4 pN/s). These curves are analyzed using the WLC model to determine TL, validated against conventional TRF results (2.5 ± 0.9 kb vs. 2.7 ± 2.3 kb) (Li *et al.* 2021).

The method’s versatility lies in its adaptability for protein-DNA interaction studies. High-resolution force-extension traces reveal real-time binding/dissociation dynamics of telomere-binding proteins (*e*.*g*., TRF1 dissociates in ~20 seconds), while single-molecule manipulation quantifies binding energies (*e*.*g*., TRF1’s 11 *k*_*B*_T energy) (Li *et al.* 2021). The platform supports integration with fluorescence microscopy for multimodal analysis and enables drug discovery by testing compounds that modulate telomere-protein interactions. This streamlined approach balances technical rigor with accessibility for broader applications in telomere biology.

## PERSPECTIVE

TL is a promising biomarker for age-associated diseases and cancer. Single-molecule studies of human TL are advancing rapidly, offering unprecedented insights into the dynamics and variability of telomeres at the single-molecule level. These methods have the potential to overcome the limitations of traditional techniques, providing a simple, rapid, and scalable approach for TL analysis. As the accuracy and reliability of single-molecule sequencing continue to improve, their diagnostic utility in predicting cellular senescence and age-related diseases will likely expand. Furthermore, the detailed information these methods provide on single telomeres will be crucial for understanding the mechanisms underlying telomere maintenance and dysfunction, and for developing targeted therapies for conditions such as short telomere syndromes and cancer.

Future research should focus on optimizing single-molecule techniques to enhance their sensitivity and throughput, making them more accessible for routine clinical use. Additionally, integrating single-molecule TL data with other genomic and molecular markers will help refine risk stratification and personalized treatment strategies. The potential of targeting telomere maintenance pathways, particularly through the inhibition of shelterin, in cancer therapy is also highly promising and warrants further investigation. Overall, single-molecule studies of human TL are poised to play a pivotal role in advancing our understanding and clinical management of age-associated diseases and cancer.
